# A New Proposal for Adequate Resection Margins in Larynx and Hypopharynx Tumor Surgery—Are the RCP Guidelines Feasible?

**DOI:** 10.3390/cancers16112058

**Published:** 2024-05-29

**Authors:** Simone E. Bernard, Cornelia G. F. van Lanschot, Jose A. Hardillo, Dominiek A. Monserez, Cees A. Meeuwis, Robert J. Baatenburg de Jong, Senada Koljenović, Aniel Sewnaik

**Affiliations:** 1 Department of Otorhinolaryngology and Head and Neck Surgery, Erasmus MC Cancer Institute, University Medical Center Rotterdam, P.O. Box 2040, 3000 CA Rotterdam, The Netherlands; 2 Department of Pathology, Erasmus MC, University Medical Center Rotterdam, P.O. Box 2040, 3000 CA Rotterdam, The Netherlands; 3 Department of Pathology, Antwerp University Hospital, Drie Eikenstraat 655, B-2650 Edegem, Belgium; 4 Faculty of Medicine, University of Antwerp, Universiteitsplein 1, B-2610 Antwerpen, Belgium

**Keywords:** laryngeal cancer, hypopharyngeal cancer, resection margins, squamous cell carcinoma, Royal College of Pathologists

## Abstract

**Simple Summary:**

A total laryngectomy and/or pharyngectomy is an important therapy modality for advanced primary and recurrent laryngeal and hypopharyngeal squamous cell carcinoma. The surgical margins are an important prognostic factor. Resection margins for head and neck cancer surgery are clear >5 mm, close 1–5 mm and positive <1 mm. However, the anatomy of the larynx and hypopharynx is complex and resections are constrained by the anatomical relationship with the surrounding structures. The aim of this study is to investigate if a margin >5 mm is feasible. Clear resection margins are not always feasible in some resection surfaces in laryngeal and hypopharyngeal surgery, due to the anatomy of the larynx and tumor location. However, striving for a maximum feasible margin is still the main goal. We propose a new guideline for the maximum feasible but adequate resection margins in larynx and hypopharynx tumor surgery.

**Abstract:**

Background: Resection margins are an important prognostic factor for patients with head and neck cancer. In general, for head and neck surgery, a margin >5 mm is advised by the Royal College of Pathologists. However, this cannot always be achieved during laryngeal and hypopharyngeal surgery. The aim of this study is to identify the resection surfaces and measure the maximum feasible margins per subsite. The clinical relevance of these maximum feasible resection margins were analyzed in this descriptive anatomical study. Methods: head and neck surgeons and a pathologist from the Erasmus MC performed a total laryngectomy and laryngopharyngectomy on a head and neck specimen specifically available for research. Results: For a total laryngectomy, resection margins >5 mm were not feasible for the ventral and dorsal resection surface. For a total laryngopharyngectomy, resection margins >5 mm were not feasible for the ventral, dorsal and lateral resection surface. Conclusion: Clear resection margins, defined as a margin >5 mm, are not always feasible in laryngeal and hypopharyngeal surgery, due to the anatomy of the larynx and tumor location. However, striving for a maximum feasible margin is still the main goal. We propose a new guideline for maximum feasible but adequate resection margins in larynx and hypopharynx tumor surgery.

## 1. Introduction

One of the most common head and neck cancers is laryngopharyngeal squamous cell carcinoma [1]. For these tumors, surgery remains an important modality, next to organ preserving (chemo)radiation techniques. The goal of surgery is to achieve complete tumor removal with clear margins, as this is one of the crucial prognostic factors for head and neck cancer [2,3]. Resection margins (i.e., the distance between tumor border and resection surface) are defined by the Royal College of Pathologists (RCP) as follows: clear >5 mm, close 1–5 mm and positive <1 mm, without distinguishing subsites in the head and neck area. Margins containing severe dysplasia or in situ carcinoma are classified as positive [4]. The guidelines of the RCP, providing textual guidance and reporting preformats, are created to assist pathologists and facilitate accurate cancer staging [4].

Although >5 mm margins can mostly be achieved during tongue excisions, for larynx and hypopharynx resections, obtaining such margins can be a challenge. The anatomy is complex and resection margins are limited by the surrounding critical structures like skin, prevertebral structures and vital neurovascular structures. The larynx and hypopharynx could be seen as a tubular organ surrounded by musculature, cartilage and fascia. Tumors grow towards the lumen, into the wall of the tubular organ towards surrounding structures, or cranial and caudal from the starting point of the tumor. Thus, resections are constrained by the anatomy and limited thickness of the different tissue layers. A balance between achieving >5 mm margins and sparing healthy tissue to maintain function and aesthetics is essential.

There is limited evidence supporting the clinical relevance of resection margins as defined by the RCP in laryngeal and hypopharyngeal cancer. Moreover, published studies did not show a significant association of >5 mm margins and overall survival (OS) (*p* = 0.93) nor with disease free survival (DFS) (*p* = 0.71) [5]. Also, resection margins are not an independent predictor for OS (*p* = 0.11) [6] or recurrence in uni- or multivariate analysis [7]. However, surgeons will always strive for clear margins because a positive margin worsens the prognosis [8,9]. Although a margin of >5 mm (according to the RCP) is the goal in larynx/hypopharynx surgery, a question remains as to whether this margin is always feasible. The aim of this study is to investigate if a margin >5 mm is feasible, per subsite.

## 2. Materials and Methods

**Figure 1:**
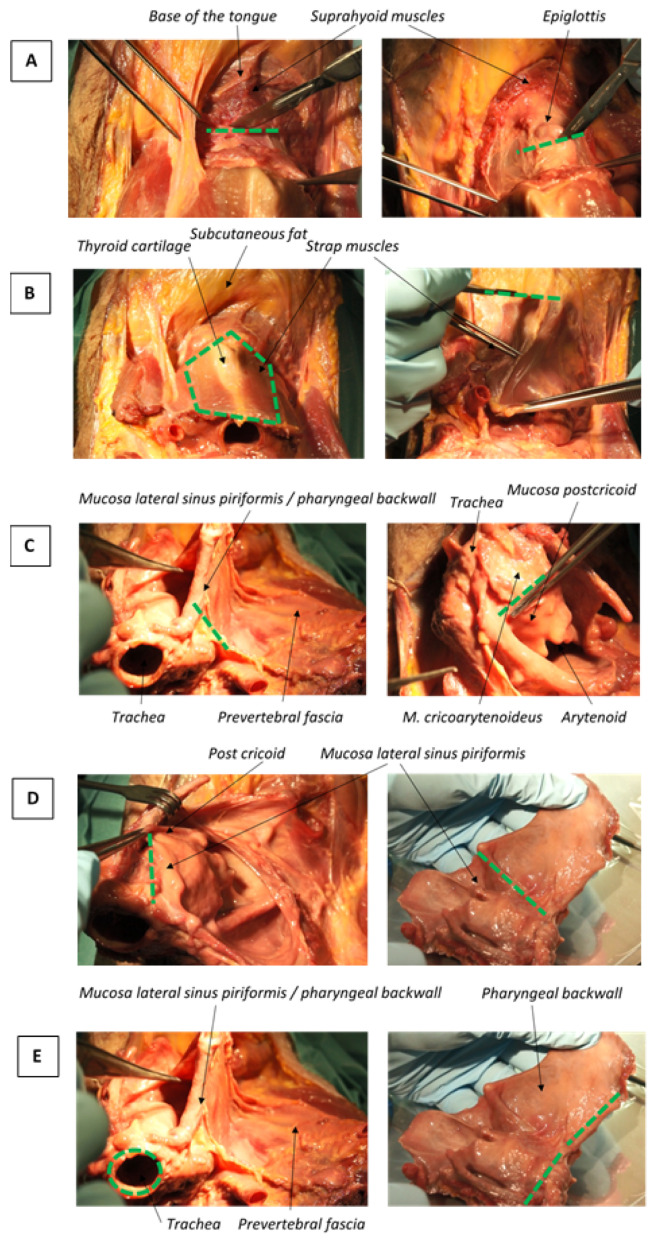
The resection surfaces per subsite and tumor location. (**A**) Cranial resection surface. (**B**) Ventral resection surface. (**C**) Dorsal resection surface. (**D**) Lateral resection surface. (**E**) Caudal resection surface.

To investigate if a resection margin >5 mm (defined according to the non-organ specific RCP guideline) is feasible in the different subsites of the larynx and hypopharynx, a panendoscopy and a total laryngectomy (TL), laryngopharyngectomy (TLP) on a fresh frozen specimen were performed. A diagnostic panendoscopy of a patient with a laryngopharyngeal squamous cell carcinoma was recorded by film and photo in order to identify the different resection surfaces of the larynx and hypopharynx. A head and neck surgeon (A.S.) and two researchers identified the different resection surfaces per subsite and tumor location. Subsequently, a team of all five head and neck surgeons of the Erasmus MC and the only dedicated head and neck pathologist (S.K.) performed a TL and TLP on one intact fresh frozen head and neck specimen, specifically available for research, at the Erasmus MC skills lab. This procedure was also recorded by film and photo. During the resection, the resection surfaces per subsite and tumor location were described and measured (Figure 1). The maximum feasible resection margins (MFM) were defined as resection margins limited to 1–5 mm based on the anatomy and limited thickness of the different tissue layers at the resection surface. The MFMs were determined per tumor location and subsite in the larynx (i.e., supraglottic, glottic and subglottic) and hypopharynx (i.e., piriform sinus, postcricoid and posterior pharyngeal wall). Afterwards, the transcript of the resections and agreed MFM were discussed with the same team of head and neck surgeons and pathologist. This study was approved by the Medical Ethics Committee (MEC-2017-336).

## 3. Results

### 3.1. Resection Surfaces and Maximum Feasible Margins (MFM) for a Total Laryngectomy (Tumors Located Supraglottic, Glottic and Subglottic)

1. Cranial. The cranial resection surface includes the suprahyoidal muscles (m. digastricus, m. geniohyoideus, m. mylohyoideus, m. stylohyoideus and m. styloglossus) and base of the tongue. A resection margin of >5 mm is feasible for all subsites.

2. Caudal. The caudal resection surface is the trachea. A resection margin of >5 mm is feasible for all subsites.

3. Ventral. The ventral resection surface includes the thyroid cartilage, strap muscles (m. thyrohyoid, cricothyroid and sternohyoid), the superficial layer of the deep cervical fascia and the skin. A resection margin >5 mm is feasible for endolaryngeal tumors. In cases of invasion of the thyroid cartilage a >5 mm margin is not feasible given the thickness of the strap muscles (4 mm measured intra-operative), fascia and skin. In the case of clinical invasion of the skin, an additional resection of the skin must be performed.

4. Dorsal. The dorsal resection surface includes the mucosa extending from the arytenoids to the postcricoid and esophageal inlet. A resection margin of >5 mm is not feasible for all subsites because of the thickness of the mucosa (2 mm measured intra-operative). Dorsal to this resection surface is the lumen of the hypopharynx and esophagus, and thus, air.

5. Lateral. The lateral resection surface includes the mucosa of the piriform sinus. A resection margin of >5 mm is feasible for all subsites.

### 3.2. Resection Surfaces and Maximum Feasible Margins (MFM) for a Total Laryngopharyngectomy (Tumors Located in the Piriform sinus, Postcricoid or Posterior Pharyngeal Wall)

1. Cranial. The cranial resection surface includes the mucosa of the lateral and posterior oropharyngeal wall, suprahyoidal muscles outside the larynx and the base of the tongue. For all subsites, a resection margin of >5 mm is feasible.

2. Caudal. The caudal resection surface is the esophagus. For all subsites, a resection margin of >5 mm is feasible.

3. Ventral. The ventral resection surface includes the thyroid cartilage, strap muscles (4 mm measured intra-operative), superficial layer of the deep cervical fascia and skin. It is of significance for anterior, medial and lateral piriform sinus tumors. The agreement regarding the resection margins for the ventral resection surface in a TL, applies here as well. The posterior pharyngeal wall tumor does not have a ventral resection surface due to the lumen of the hypopharynx at the ventral side. The postcricoid tumors have the (endo)larynx as a ventral resection surface.

4. Dorsal. The dorsal resection surface includes the hypopharyngeal mucosa, m. prevertebralis, prevertebral fascia and the vertebral column. It is relevant for lateral wall sinus piriformis tumors or posterior pharyngeal wall tumors. A resection margin of >5 mm is not feasible due to the thickness of the mucosa (<1 mm measured intra-operative) and prevertebral fascia. Invasion through the prevertebral fascia makes the tumor inoperable. A resection margin of >5 mm is feasible for anterior wall sinus piriform tumors. The medial wall piriform sinus tumor and postcricoid tumor do not have a dorsal resection surface due to the lumen of the hypopharynx and esophagus.

5. Lateral. The lateral resection surface comprises the mucosa, m. constrictor pharyngeus and carotid space with its own layer of deep cervical fascia. It is only important in the case of lateral and anterior piriform sinus tumors. A resection margin >5 mm is not feasible due to the thickness of the mucosa (3 mm measured intra-operative) and vital vascular structures directly lateral. Encasement of the carotid artery makes the tumor inoperable. A medial wall piriform sinus tumor does not have a lateral resection surface due to the lumen of the hypopharynx at the lateral side. For posterior pharyngeal wall and postcricoid tumors, a resection margin of >5 mm is feasible.

Table 1 gives an overview of the resection surfaces per tumor location. Table 2 gives an overview of the resection surfaces where >5 mm margins are not feasible.

## 4. Discussion

Although resection margins are an important prognostic factor for head and neck cancer, the question of whether the RCP guidelines can be applied in the complex anatomical area of the larynx and hypopharynx arises. The RCP guideline for mucosal malignancies of the larynx describes how to record a histopathology report whereby the diameter, depth of invasion, cartilage invasion, invasion of the deep tissue planes (paraglottic and pre-epiglottic space) and differentiation grade need to be documented. The resection margins (mucosal and deep) are defined as clear >5 mm, close 1–5 mm and positive <1 mm and are only briefly discussed. The required orientation (ventral, dorsal, etc.) or a way to measure these margins are not mentioned. Only the following is described: “deep resection margins may be inapplicable unless the tumor extends into the soft tissue of the neck or close to the base of the tongue” [4]. It is unclear which resection surfaces or resection margins are implied. Also, the guidelines of the RCP are not organ specific. The authors explain that incomplete resection or the presence of dysplasia at the margin is associated with a significantly increased risk of local recurrence. However, they refer to four articles which describe the clinical relevance of resection margins for all the head and neck subsites [4]. There is lack of evidence for resection margins in the larynx and hypopharynx. As mentioned earlier, the anatomy of the subsites in the head and neck area is different and the clinical importance of a >5 mm margin per subsite cannot be compared.

The question remains: is a margin of >5 mm feasible in the larynx and hypopharynx? Several studies assert that the anatomy of the head and neck region restricts resection margins due to the limited thickness of the different tissue layers [10,11,12]. In this study we show that a >5 mm margin is not always feasible due to the complex anatomy and surrounding structures. We suggest that a margin of 1–5 mm should be accepted in specific cases. Self-evidently, this needs to be justified by oncological outcome data which will be the aim of our next study.

First to be discussed is the ventral resection surface for laryngeal tumors. In the case of the invasion of the thyroid cartilage, a >5 mm margin is not feasible because the strap muscles are <5 mm thick. We do not perform an additional resection of the skin because of the related morbidity. Also, the superficial layer of the deep cervical fascia ventral to the strap muscles could be seen as a natural barrier against tumor spread. Only in the case of tumor invasion of the skin do we recommend a resection of the skin, which is also recommended in literature [13]. A second resection surface to be discussed, is the dorsal surface for laryngeal tumors. In this case, the resection surface is the mucosa extending from the posterior commissure to the arytenoids and postcricoid, but the mucosa is <5 mm thick and the hypopharyngeal lumen is dorsal to the larynx. We recommend performing an additional resection of the mucosa extending to the esophageal inlet (caudal) only if the postcricoid mucosa is invaded. In the case of no tumor invasion, an additional resection of the postcricoid mucosa is not necessary. Sparing the pharyngeal mucosa makes the primary closure of the pharynx feasible.

For hypopharyngeal tumors, the lateral resection is challenging because the constrictor muscle is <5 mm. Also, the carotid artery and internal jugular vein are lateral to these muscles. In the case of the encasement of the carotid space we consider the tumor inoperable. The dorsal resection surface for posterior pharyngeal wall tumors and lateral piriform sinus tumors are limited by the thin mucosa of the posterior pharyngeal wall, the m. prevertebralis and vertebral column. The prevertebral fascia can be seen as a natural tumor barrier. If there is invasion of the prevertebral fascia, we consider the tumor inoperable. At our institute the anatomical restrictions are respected in regard to the related morbidity for the patient. Extensive resections, for example removing the skin with an associated reconstruction, results in more morbidity with a worse functional and aesthetic outcome. The question of whether removing healthy tissue is really necessary for a better oncological outcome remains. We will always strive for a wide resection margin. However, if there is less than 5 mm space, a margin of >5 mm cannot be achieved.

Because of the complexity of the area, knowledge of the anatomy is important for the pathologist. An adequate resection is different for the surgeon and pathologist. The surgeon strives for a complete resection with maximum feasible margins, with preservation of normal tissue and function. The pathologist prefers a complete resection with >5 mm margins. Communication between the surgeon and pathologist is necessary to understand which resection surfaces are crucial. False negative resection margins could result in undertreatment (i.e., missing out on adjuvant therapy) and false positive resection margins could result in overtreatment and unnecessary concern. We propose to perform an intra-operative assessment of resection margins on the specimen where the surgeon and pathologist together assess the resection specimen visually, by palpation and by making incisions perpendicular to the resection plane to accurately record the resection surfaces of importance and determine the MFM.

Moreover, we designed a template for the structured registration of margins of the larynx and hypopharynx resection specimen during the surgicopathological evaluation (Table 3). Also, for other head and neck subsites, such as the oropharynx, mandibular or maxillary region, it can be difficult to achieve clear margins (>5 mm) [14], and the same consideration applies for these tumor locations.

In this study we performed our measurements during a diagnostic panendoscopy and on a fresh-frozen specimen to achieve the most accurate measurements of the resection margins. A limitation of this study is that the dissection was performed on one fresh-frozen specimen and measurements were taken intra-operatively during a TL and TLP. The thickness of different laryngeal structures could vary per patient. In literature, shrinkage of the head and neck cancer specimens and margin dimensions are described due to intrinsic tissue properties and formalin effects. It is therefore recommended to measure immediately after the resection or during the intra-operative assessment to avoid underestimation of the resection margins [15,16,17]. As mentioned before, it is our recommendation that the surgeon and the pathologist should carefully document the measurements of the resection margins during intra-operative assessment.

Currently, it is unclear whether there is a universal approach following the guidelines of the RCP or if resection margins of 1–5 mm, based on the restrains of the anatomy, are accepted. An extensive literature search in Medline, Embase and the Cochrane Collaboration showed a lack of studies regarding the clinical relevance of resection margins in the larynx and hypopharynx. Twelve articles regarding resection margins during TL/TLP were found. A single article followed the RCP guidelines [9]. Two studies defined close margins as <5 mm and positive margins as situations in which a tumor is present at the resection surface [18,19]. The remaining nine articles used descriptive definitions for margin status, instead of the exact value of the resection margin, such as ‘positive’, ‘microscopically positive’, ‘tumor at the resection surface’, ‘negative’, ‘safe margins’ or ‘no invasive tumor at the resection surface’. It is therefore not possible to clarify the clinical relevance of the resection margins in the larynx and hypopharynx. Saraniti et al. confirms this by stating ‘To reach a unanimous agreement regarding the prognostic value of resection margins, it would be necessary to carry out meta-analyses on studies sharing a definition of resection margin, methodology and post-operative therapeutic choices’ [7]. In order to introduce the MFM for laryngeal and hypopharyngeal cancer to the guidelines of the RCP and implement this as a standard of care, it is important to investigate its clinical relevance. Our next study will focus on recurrence rates and survival data regarding the resection margins. Further surgically and histopathologically oriented studies are recommended to thoroughly describe the anatomy of the critical areas for surgical margins.

## 5. Conclusions

To our knowledge, this is the first study to investigate and describe the maximum feasible resection margins for all resection surfaces in a total laryngectomy and laryngopharyngectomy. This study challenges the RCP guidelines after showing that resection margins of >5 mm are not always feasible at every subsite in the larynx and hypopharynx. We advocate maximum feasible margins of >1 mm, instead of >5 mm, to enable an adequate resection. The focus of our next study will be to justify this proposal with oncological outcome data.

## Figures and Tables

**Table 1:** Resection surfaces per tumor location from inside to outside.

Tumor Location	Surgery	Cranial	Caudal	Ventral	Dorsal	Lateral
Larynx - supraglottic - glottic - subglottic	TL	suprahyoidal muscles -> tongue base	trachea	thyroid cartilage -> strap muscles -> superficial fascia -> skin	mucosa postcricoid and lumen hypopharynx and esophagus	mucosa piriform sinus
Piriform sinus - lateral (L) - anterior (A) - medial (M)	TLP	lateral oropharyngeal mucosa -> suprahyoidal muscles -> tongue base	esophagus	thyroid cartilage -> strap muscles -> superficial fascia -> skin	- L: hypopharyngeal mucosa - A: medial and lateral piriform sinus -> lumen hypopharynx - M: lumen hypopharynx (no resection surface)	- L: m. constrictor pharyngeus -> internal jugular vein -> carotid artery - A: m. constrictor pharyngeus -> internal jugular vein -> carotid artery - M: lumen hypopharynx (no resection surface)
Postcricoid	TLP	(endo)larynx	esophagus	(endo)larynx	lumen hypopharynx (no resection surface)	mucosa piriform sinus
Posterior pharyngeal wall	TLP	posterior oropharyngeal mucosa	esophagus	lumen hypopharynx (no resection surface)	m. prevertebralis -> prevertebral fascia -> vertebrae	lateral hypopharyngeal mucosa

**Table 2:** Resection surfaces where >5 mm margins are not feasible in total laryngopharyngectomy.

Tumor Location	
Larynx (supraglottic, glottic and subglottic)	Ventral (4 mm measured intra-operative): In the case of invasion of the cartilage. In the case of invasion of the skin, additional resection is needed Dorsal (2 mm mucosa postcricoid measured intra-operative): n.a. (lumen of the hypopharynx and esophagus)
Piriform sinus Lateral (L)	Ventral (4 mm measured intra-operative): In the case of invasion of the cartilage. In the case of invasion of the skin, additional resection is needed Dorsal (<1 mm measured intra-operative): prevertebral fascia Lateral (3 mm measured intra-operative): carotid space
Anterior (A)	Ventral (4 mm measured intra-operative): In the case of invasion of the cartilage. In the case of invasion of the skin, additional resection is needed Lateral (3 mm measured intra-operative): carotid space
Medial (M)	Ventral (4 mm measured intra-operative): In the case of invasion of the cartilage. In the case of invasion of the skin, additional resection is needed Dorsal: n.a. (lumen hypopharynx) Lateral: n.a. (lumen hypopharynx
Postcricoid	Dorsal: n.a. (lumen hypopharynx)
Posterior pharyngeal wall	Ventral: n.a. (lumen hypopharynx) Dorsal (<1 mm measured intra-operative): prevertebral fascia

**Table 3:** Pathology report with resection margins in millimeters.

Laryngeal tumors		Cranial Suprahyoidal muscles– tongue base	Caudal Trachea	Ventral ^1^ Thyroid cartilage–strap muscles–fascia–skin	Dorsal ^2^ Mucosa arytenoids to postcricoid	Lateral Mucosa piriform sinus
	Supraglottic	… mm	… mm	… mm **	… mm *	… mm
	Glottic	… mm	… mm	… mm **	… mm *	… mm
	Subglottic	… mm	… mm	… mm **	… mm *	… mm
Hypopharyngeal tumors		Cranial Mucosa oropharynx – suprahyoidal muscles– tongue base	Caudal Esophagus	Ventral ^1^ Larynx	Dorsal ^2^ Mucosa hypopharynx– m. prevertebralis–fascia–vertebral column	Lateral Mucosa–m. constrictor pharyngeus–vessels
Medial wall piriform sinus		… mm	… mm	… mm **	n.a. (lumen hypopharynx)	n.a. (lumen hypopharynx)
Anterior wall piriform sinus		… mm	… mm	… mm **	… mm	… mm *
Lateral wall piriform sinus		… mm	… mm	… mm **	… mm *	… mm *
Postcricoid		… mm	… mm	… mm	n.a. (lumen hypopharynx)	… mm
Posterior pharyngeal wall		… mm	… mm	n.a. (lumen hypopharynx)	… mm *	… mm

^1^ Extralaryngeal growth and/or resection of the skin performed. ^2^ Invasion of m. cricoarytenoideus/mucosa postcricoid. * >5 mm not possible due to anatomy. ** >5 mm not possible in the case of invasion of the thyroid cartilage. … Space for editing the measured resection margins in mm.

## Data Availability

The data presented in this study are available in this article.
